# High stromal nicotinamide N‐methyltransferase (NNMT) indicates poor prognosis in colorectal cancer

**DOI:** 10.1002/cam4.2890

**Published:** 2020-01-27

**Authors:** Mengmeng Song, Ye Li, Mingyong Miao, Fan Zhang, Hao Yuan, Fuao Cao, Wenjun Chang, Hanping Shi, Chunhua Song

**Affiliations:** ^1^ Department of Epidemiology and Statistics College of Public Health Zhengzhou University Zhengzhou China; ^2^ Department of Digestive Endoscopy Shuguang Hospital Shanghai University of Traditional Chinese Medicine Shanghai China; ^3^ Department of Biochemistry Second Military Medical University Shanghai China; ^4^ Department of Environmental Health Second Military Medical University Shanghai China; ^5^ Department of Colorectal Surgery Changhai Hospital Second Military Medical University Shanghai China; ^6^ Department of Gastrointestinal Surgery/Clinical Nutrition Beijing Shijitan Hospital Capital Medical University Beijing China

**Keywords:** clinical significance, colorectal cancer, NNMT, prognosis

## Abstract

**Purpose:**

Nicotinamide n‐methyltransferase (NNMT) has good biochemical activity and epigenetic regulation, and has been reported as a major metabolic regulator of cancer. The goal of this study was to investigate the significance of stromal NNMT expression in colorectal cancer (CRC).

**Patients and methods:**

Stromal expression of NNMT in primary CRC, metastasis CRC, and their non‐cancerous tissues from 1088 CRC patients was examined by immunohistochemistry. The associations between stromal NNMT expression and survival outcomes in 967 patients with stage I‐III CRC were further evaluated with Kaplan‐Meier curve and Cox model analyses.

**Results:**

NNMT expression was mainly sourced from stromal compartments and also elevated in CRC. Patients with high stromal NNMT (IHC‐score ≥ 106) have a worse survival than those patients with low stromal NNMT. In multiple Cox analyses, high expression of stromal NNMT remained as an independent risk factor in CRC for disease‐free survival with a hazard ratio (HR) of 1.415 (95% confidence interval [CI], 1.015‐1.972) and disease‐specific survival with a HR of 5.004 (95% CI, 2.301‐10.883). In addition, high stromal NNMT expression in CRC also indicates the poor survival outcomes in patients with early stage CRC (stage I and II) and in patients who undergo chemotherapy.

**Conclusion:**

NNMT is mainly located in CRC stromal compartment. High stromal NNMT expression predicts an unfavorable postoperative prognosis.

## Graphical abstract

Our study identified the mainly sources of NNMT in CRC and reports firstly the clinical significance of stromal NNMT in CRC. Currently, little progress has been made with regard to the prognosis and treatment for CRC. Our findings are of great importance in the prognostic classification and the founding of new candidates of CRC.

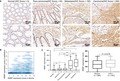

## 1 INTRODUCTION

Nicotinamide N‐methyltransferase (NNMT) is a known enzyme that catalyzes the pyridine compounds and N‐methylation of nicotinamide.^1^, ^2^ NNMT transfers a reactive methyl group of S‐adenosyl methionine (SAM) to nicotinamide to form the metabolically inert product 1‐methyl nicotinamide (1‐MNA) and S‐adenosyl homocysteine (SAH).^2^ SAM is a universal methyl donor of various molecules, such as histone, nonhistone, DNA, and other metabolites.^3^ This activity produces a methyl sink in the form of 1‐MNA, leading to depletion of SAM and reduces the overall methylation potential of cells. NNMT mediates SAM depletion and attenuating histone methylation in various cells, including cancer cells,^4^ which resulted in the extensive gene expression changes in the tumor stroma. Besides, NNMT can promote the CRC cell growth and increase the resistance of CRC cells to 5‐fluorouracil through the 1‐MNA production.^5^, ^6^ High expression of NNMT has been reported in multiple cancer types.^7^, ^8^, ^9^, ^10^, ^11^, ^12^ Several studies have shown that the elevated NNMT level in cancer can be used for the early diagnosis and noninvasive diagnosis of some malignant tumors.^13^, ^14^, ^15^ Interestingly, NNMT has been considered as a novel and sensitive serum biomarker for CRC detection, outperforming the clinical significance of carcinoembryonic antigen (CEA).^12^ However, it was recently reported that NNMT was expressed in stroma and served as a major metabolic regulator of ovarian cancer‐associated fibroblasts (CAF).^4^ The tumor microenvironment is critical in the development and progression of CRC, and stromal signatures in different tumor types have been described in the earlier works with possible clinical significance.^4^, ^16^ However, the relevant markers, especially key regulators, have been scarcely reported. NNMT is also expressed in the stroma of colorectal tissue,^4^ but the clinical significance of the protein is unknown. In this study, the expression of stromal NNMT with immunohistochemistry (IHC) on tissue microarrays (TMAs) containing tissue dots from 967 patients with CRC and 121 CRC liver metastases were examined. Then, the association between NNMT expression and clinical features and patient survival was analyzed. We found that high expression of stromal NNMT was significantly associated with advanced TNM stages, poor prognosis, and chemotherapy resistance in patients with CRC. The evidence above indicated that stromal NNMT may serve as a promising prognostic biomarker in CRC.

## 2 MATERIALS AND METHODS

### 2.1 Bioinformatics

The processed data from TCGA‐CRC were downloaded from the cBioPortal website and contained 592 CRC tissues for further analysis. Raw data from 2 datasets (http://www.ncbi.nlm.nih.gov/geo/query/acc.cgi?acc=GSE39582 and http://www.ncbi.nlm.nih.gov/geo/query/acc.cgi?acc=GSE35144) were downloaded from the National Center for Biotechnology Information (NCBI) Gene Expression Omnibus (GEO) repository (http://www.ncbi.nlm.nih.gov/geo). Each dataset was based on the Affymetrix plus 2.0 platforms (Santa Clara, CA, USA), and the corresponding gene profiles was extracted with the fRMA package^17^, ^18^ in the R 3.2.0 environment. The coexpression pattern of NNMT with known markers of cancer‐associated fibroblasts (CAFs), such as ACTA2 and POSTN, were analyzed in the datasets of TCGA‐CRC and http://www.ncbi.nlm.nih.gov/geo/query/acc.cgi?acc=GSE39582 datasets with Pearson correlation coefficients at a value of *P* < .05. The http://www.ncbi.nlm.nih.gov/geo/query/acc.cgi?acc=GSE35144 dataset, containing the transcriptional profiles of 28 human CRC tissues and 37 patient‐derived CRC explants (PDCCE), was also were used to verify the compartments of NNMT expression.

### 2.2 Patients and specimens

Formalin‐fixed, paraffin‐embedded (FFPE) tissues from 967 cancerous, 34 adenoma, and 38 precancerous were obtained from 967 CRC patients who underwent surgical resection at the Changhai Hospital from January 2006 to November 2011. Patients receiving preoperative chemotherapy or radiation were excluded from the study. The clinical characteristics of each patient, such as age, sex, tumor location, number of resected lymph nodes, differentiation grade, TNM staging (according to the American Joint Committee on Cancer Staging System, 7th edition), adjuvant chemotherapy, and serum carcinoembryonic antigen (CEA), and carbohydrate antigen 199 (CA199) levels were collected. FFPE tissues from 121 CRC liver metastases and 37 normal colorectal tissues were also collected in the hospital. The follow‐up information of the 967 CRC patients were collected as described in a previous study.^19^ The main outcomes of interest were disease‐specific survival (DSS) and disease‐free survival (DFS). The number of months from the date of receiving surgery to the date that the patient died of CRC and the number of months from the date of receiving surgery to the date of the first relapse defined as DSS and DFS, respectively. This study was approved by the Institutional Review Committee of Changhai Hospital. All participants signed a written informed consent form, giving permission for the biomaterials to be used in the study.

### 2.3 Immunohistochemistry

Tissue microarrays (TMAs) containing the FFPE specimens were constructed by a commercial agent (Outdo Biotech, Shanghai, China) and then used to examine the expression pattern of stroma NNMT via using immunohistochemistry (IHC). The examination of IHC was processed according to a standard procedure with 4‐µm‐thick sections. In Brief, all array slides were first baked overnight at 60°C, deparaffinized using xylene and rehydrated using graded ethanol series. The endogenous peroxidase was blocked for 5 minutes with 3% H_2_O_2_. The NNMT antigen was retrieved by placing all slides in boiling sodium citrate (10 mmol/L, pH 6.0, at 100℃) for 30 minutes. Then, each slide was incubated overnight at 4℃ with rabbit anti‐human NNMT polyclonal antibody (1:1000, HPA059180; Sigma‐Aldrich Co.) according to the manufacturer's instructions. The specificity of the antibody has been verified, and the corresponding data can be found at the web of The Human Protein Atlas website (https://www.proteinatlas.org/). After incubation for 30 minutes with secondary antibodies from the Elivision^TM^super HRP (Mouse/Rabbit) IHC Kit (kit‐9922; Maxvision, Foshan, People's Republic of China), each slide was reacted with 3‐3‐aminobenzidine (DAB) solution for 45 seconds and stained with hematoxylin for 25 seconds. All slides were stained simultaneously by the same individual researcher to eliminate intraassay variation.

### 2.4 Quantitative evaluation of stromal NNMT immunostaining

The stained TMA slides were scanned by the Servicebio system (digital scanning via Pannoramic MIDI; 3Dhistech, Budapest, Hungary, Europe), and CaseViewer software was used to evaluate the images under a bright‐field microscopy at resolution of × 200. The *H*‐score method 20 was used to quantify the NNMT protein. The immunostaining intensities of stroma NNMT expression were assessed by measuring the percentage of fibroblasts that were negative (0), weakly positive (1+), moderately positive (2+), and strongly positive (3+), and we also calculated the percentage of intensity staining for each specimen. The *H*‐score was calculated by multiplying the average percentage of positive cells by the corresponding staining intensity (*H*‐score range: 0‐300). The assessment of all slides was evaluated by two independent investigators (MS and YL) who were blinded to the clinical pathology information. The difference between the observers was averaged.

### 2.5 Statistical analysis

Using paired Student t‐test to evaluate the discrimination power of the IHC scores of stromal NNMT for paired cancer and normal tissues. Independent sample t‐test was used to compare the differences of IHC scores of stromal NNMT between CRC specimens. A descriptive summary of the clinicopathological features was performed using independent Student *t* test (for continuous variables) and Pearson Chi‐square test (for categorical variables). The Maxstat package was used to identify the optimal cut‐off value of the IHC score to define risk subgroups.^21^ The Kaplan‐Meier curves were used to estimate the DSS and DFS, and the survival curves was compared by the log‐rank test. The hazard ratios (HRs) and corresponding 95% confidence intervals (CIs) were estimated by Cox proportional hazards models. All statistical tests were two‐sided and performed by SPSS (Version 21.0 for Windows) and R 3.5.1. *P* < .05 was considered statistically significant.

## 3 RESULTS

### 3.1 Elevated NNMT expression in the stromal compartment of CRC tissues

**Figure 1:**
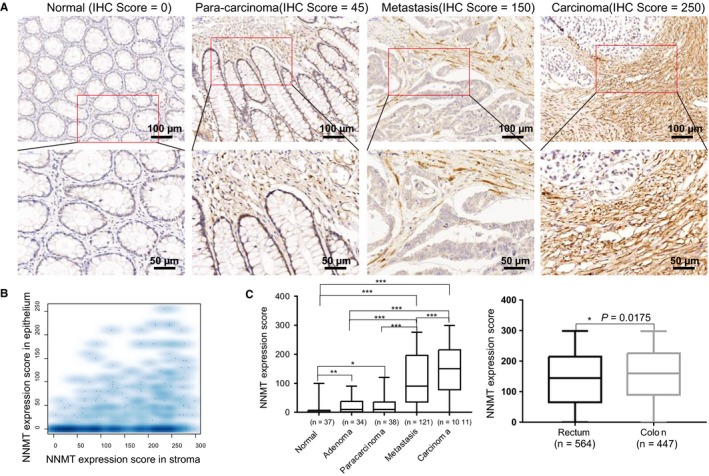
NNMT protein is elevated in colorectal cancer stroma and epithelium. A, Representative examples of immunostaining of NNMT in CRC and adjacent normal tissues, adenoma tissues, paracarcinoma tissues and metastasis tissues. Bar, 50 µm. B, IHC‐score scatterplot (R software) of stomal NNMT and epithelial NNMT. NNMT is mainly expressed in the cytoplasm of colorectal stromal cells. (color density representation of a scatterplot) C, Expression pattern of NNMT protein in formalin‐fixed paraffin‐embedded specimens of CRC and adjacent normal tissues, adenoma tissues, paracarcinoma tissues and metastasis tissues. The differences of NNMT protein expression between colon cancer vs rectum cancer. *P < .05; **P < .01; ***P < .001

To explore the compartments of NNMT expression in CRC, we first employed gene correlation analysis on the TCGA‐CRC dataset (n = 594) using the website of cBioportal website. The results showed that the expression of NNMT mRNA was significantly highly correlated with many stromal marker genes, such as ACTA2, VIM, FAP, SPARC, POSTN, and TAGLN (Figure S1A). The results were further verified with another CRC transcriptional profiling dataset with the GSE number of http://www.ncbi.nlm.nih.gov/geo/query/acc.cgi?acc=GSE39582 (n = 585) (Figure S1A). We also found that the expression of NNMT was elevated by more than fivefold change in human CRC tissues than the tissues in PDCCE models (Figure S1B), which further indicated that NNMT may be expressed mainly in the stromal compartment of CRC. The examination of NNMT by using IHC in our cohorts showed that the protein was mainly located in the stromal cells or cytoplasm of epithelial cells in colorectal cancerous or noncancerous tissues (Figure 1A). Although previous studies reported that the protein expressed in the CRC cells as an oncogene, our results showed that the stromal compartments in CRC contained more NNMT proteins than cancerous epithelial compartments (Figure 1B).

Moreover we compared the expression pattern of stromal NNMT in different tissues including CRC tissues, adjacent normal tissues, adenoma, paracarcinoma tissues, and CRC liver metastases. The results showed that stromal NNMT expression in CRC tissues and CRC liver metastases were significantly higher as compared with normal, paracarcinoma and adenoma tissues (*P* < .01; Figure 1C). Notably, the expression of stromal NNMT increased gradually among in normal, adenomas, and cancerous tissues (all *P* < .05; Figure 1C). The expression differences in stromal NNMT were also found to be significantly between colon and rectal cancerous tissues (*P* < .05; Figure 1C).

### 3.2 Associations between stromal NNMT and patient features

According to the 97.5% quantile IHC‐score of noncancerous specimens (n = 108), the 967 CRC specimens of patients with CRC were first classified into the subgroups with high (IHC score ≥ 106) or low (IHC score < 106) stromal NNMT expression. Then, the associations between the status of stromal NNMT and clinicopathological characteristics were analyzed in CRC patients. As shown in Table 1, higher expression of stromal NNMT was significantly associated with advanced TNM stage (*P* = .043), which remained significant only in rectal cancer but not in colon cancer and was marginally associated with serum CA199 in CRC (*P* = .063). In addition, higher stromal NNMT expression was also significantly associated with poor tissue differentiation only in rectal cancer (*P* = .028). Due to the associations between the patient survival and TNM stages and differentiation grade, the results above indicated that stromal NNMT may contribute to the progression of CRC, although the inconsistencies between stromal NNMT and some clinical features were existed between colon and rectal cancer.

**Table 1:** Characteristics of patient with CRC dichotomized by NNMT expression

Variables	All (n = 967)			Colon (n = 457)			Rectum (n = 510)		
	NNMT^L^ (n = 326)	NNMT^H^ (n = 641)	*P* value	NNMT^L^ (n = 153)	NNMT^H^ (n = 304)	*P* value	NNMT^L^ (n = 173)	NNMT^H^ (n = 337)	*P* value
Age, mean ± SD	60.89 ± 11.90	60.80 ± 12.70	.919	61.09 ± 12.89	62.46 ± 13.56	.299	60.72 ± 10.99	59.31 ± 11.69	.189**
Sex, n (%)									
Male	190 (58.3)	383 (59.8)	.660	79 (51.6)	170 (55.9)	.385	111 (64.2)	213 (63.2)	.832*
Female	136 (41.7)	258 (40.2)		74 (48.4)	134 (44.1)		62 (35.8)	124 (36.8)	
Differential grade, n (%)									
Well	11 (3.4)	8 (1.2)	.464	5 (3.3)	7 (2.3)	.295	6 (3.5)	1 (0.3)	
Moderate	288 (88.3)	585 (91.3)		130 (85.5)	276 (90.8)		158 (91.3)	309 (91.7)	**.028** ***
Poor	26 (8.0)	48 (7.5)		17 (11.2)	21 (6.9)		9 (5.2)	27 (8.0)	
Missing	1 (0.3)			1 (0.7)					
Lymph nodes, n (%)									
<12	71 (21.8)	131 (20.4)	.627	30 (19.6)	73 (24.0)	.287	41 (23.7)	58 (17.2)	.079*
≥12	255 (78.2)	510(79.6)		123 (80.4)	231 (76.0)		132 (76.3)	279 (82.8)	
TNM stage, n (%)									
I	49 (15.0)	97 (15.1)	**.043**	10 (6.5)	25 (8.2)	.661	39 (22.5)	72 (21.4)	**.027** ***
II	181 (55.5)	300 (46.8)		87 (56.9)	157 (51.6)		94 (54.3)	143 (42.4)	
III	96 (29.4)	244 (38.1)		56 (36.6)	122 (40.1)		40 (23.1)	122 (36.2)	
Chemotherapy, n (%)									
Yes	249 (76.4)	484 (75.5)	.827	113 (73.9)	228 (75.0)	.480	136 (78.6)	256 (76.0)	.295*
No	52 (16.0)	97 (15.1)		22 (14.4)	54 (17.8)		30 (17.4)	43 (12.8)	
Missing	25 (7.6)	60 (9.4)		18 (11.8)	22 (7.2)		7 (4.0)	38 (11.3)	
Serum CEA, n (%)									
<5 ng/mL	209 (64.1)	391 (61.0)	.289	96 (62.7)	170 (55.9)	.140	113 (65.3)	221 (65.6)	.979*
≥5 ng/mL	115 (35.3)	250 (39.0)		56 (36.6)	134 (44.1)		59 (34.1)	116 (34.4)	
Missing	2 (0.6)			1 (0.7)			1 (0.6)		
Serum CA199, n (%)									
<37 U/mL	286 (87.7)	537 (83.8)	.063	133 (86.9)	242 (79.6)	**.038**	153 (88.4)	295 (87.5)	.642*
≥37 U/mL	38 (11.7)	104 (16.2)		19 (12.4)	62 (20.4)		19 (11.0)	42 (12.5)	
Missing	2 (0.6)			1 (0.7)			1 (0.6)		

Abbreviations: CA199, carbohydrate antigen 199; CEA, carcinoembryonic antigen; CRC, colorectal cancer; NNMT, Nicotinamide N‐methyltransferase; NNMT^L^, NNMT low expression; NNMT^H^, NNMT high expression.

* Chi square test or Fisher's exact test. Missing values are excluded for all statistic tests.

** Student's *t* test.

*** Mann‐Whitney U test (nonparametric). Missing values are excluded for all statistic tests.

### 3.3 Stromal NNMT expression predicts unfavorable survival

**Figure 2:**
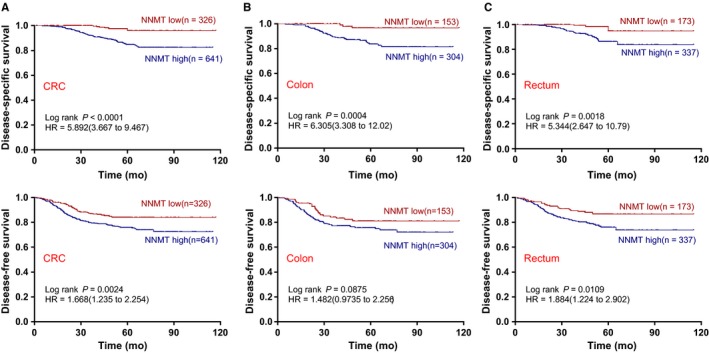
High IHC‐score of NNMT predicts poor survivals of patients with CRC. (A‐C) CRC patients, colon cancer and rectum cancer patients were dichotomised into the subgroups with NNMT‐high or NNMT‐low protein expression according to NNMT IHC‐score (cut‐off value = 106). Disease‐specific survival and disease‐free survival are presented. Log‐rank *P* values and hazard ratios (HRs) from Kaplan‐Meier analysis with log‐rank test are shown

We further investigated the prognostic value of stromal NNMT expression in CRC. Because the cut‐off value of 106 for the IHC‐score above could most effectively discriminate the differences of DFS in our CRC cohort, which was verified by Maxstat software, it was continuously used to define the patient subgroups with high stromal NNMT or low stromal NNMT. Kaplan‐Meier analysis showed that patients with high stromal NNMT were significantly associated with a poorer DFS and DSS than those CRC patients with low stromal NNMT, as shown in Figure 2A, which consistently remained in colon cancer and rectal cancer (Figure 2B,C); however, a marginal association of DFS in colon cancer was found (*P* = .087). The high expression of stromal NNMT along with TNM stage and the number of resected lymph nodes were all significantly associated with the DFS and DSS of CRC patients in univariate Cox analysis (Table 2). The variables of differentiation grade, chemotherapy treatment, and serum biomarkers were also associated with the DFS of CRC patients (Table 2). Importantly, multivariate Cox regression analysis showed that high stromal NNMT in CRC remained statistically significant predictor of DFS (HR, 1.415; 95%CI, 1.015‐1.972) and DSS (HR, 5.004; 95% CI, 2.301‐10.883) after adjusting for variables such as stage, grade, the number of resected lymph nodes, chemotherapy, serum CEA and serum CA199, as shown in Table 2.

**Table 2:** Cox regression analysis of immunohistochemistry NNMT expression and clinicopathological covariates in the CRC patients

Variables	Disease‐specific survival				Disease‐free survival			
	Univariate analysis		Multivariate analysis		Univariate analysis		Multivariate analysis	
	HR (95% CI)	*P* value	HR (95% CI)	*P* value	HR (95% CI)	*P* value	HR (95% CI)	*P* value
NNMT								
Low vs High	5.085 (2.339‐11.056)	**<.001**	5.004 (2.301‐10.883)	**<.001**	1.593 (1.145‐2.215)	**.006**	1.415 (1.015‐1.972)	**.041**
Sex								
Male vs Female	0.843 (0.534‐1.331)	.464			0.893 (0.667‐1.196)	.447		
Tumor location								
Colon vs Rectum	1.257 (0.804‐1.996)	.316			1.119 (0.894‐1.583)	.233		
Differential grade								
(Well + Moderate) vs Poor	1.251 (0.575‐2.721)	.573			1.802 (1.165‐2.787)	**.008**		
Lymph nodes, n (%)								
<12 vs ≥12	2.359 (1.269‐4.389)	**.007**	2.280 (1.230‐4.225)	**.009**	2.943 (1.848‐4.687)	**<.001**	2.954 (1.853‐4.709)	**<.001**
TNM stage								
I + II vs III	1.692 (1.084‐2.641)	**.021**			2.295 (1.725‐3.052)	**<.001**	2.049 (1.533‐2.739)	**<.001**
Chemotherapy								
Yes vs No	1.223 (0.604‐2.475)	.576			1.711 (1.034‐2.830)	**.037**		
Serum CEA (ng/mL)								
<5 vs ≥5	1.555 (0.996‐2.428)	.052			1.589 (1.195‐2.114)	**.001**	1.408 (1.041‐1.905)	**.026**
Serum CA199 (U/mL)								
<37 vs ≥37	1.448 (0.812‐2.582)	.210			1.945 (1.384‐2.733)	**<.001**	2.476 (1.026‐2.123)	**.036**

### 3.4 Stromal NNMT expression predicts an unfavorable survival in early CRC

**Figure 3:**
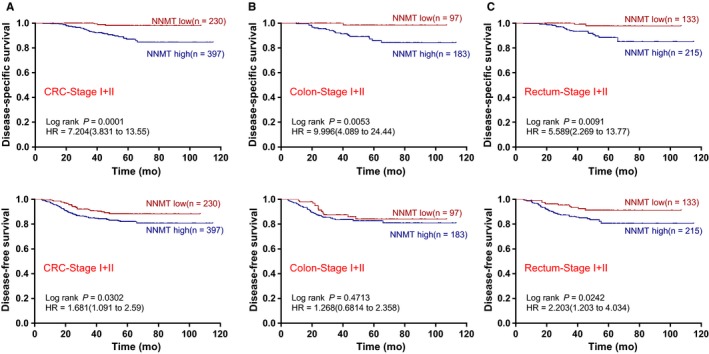
The association between stromal NNMT expression and survival of patients with earlier stage CRC (I and II stage). A, High IHC‐score of NNMT is associated with poor survivals of patients with early stage CRC. B, High NNMT expression predicts poor DSS of patients with early stage colon cancer. C, High NNMT expression is associated with poor survivals of patients with early stage rectum cancer

To further explore the relationship between stromal NNMT expression and DFS in patients early‐stage CRC (stage I and II). High stromal NNMT expression was associated with poorer DFS compared with low stromal NNMT expression in CRC and rectal cancer, and high stromal NNMT expression was associated with poorer DSS compared with low stromal NNMT expression in CRC (Figure 3A), colon cancer (Figure 3B) and rectal cancer (Figure 3C). For stage II disease alone, patients with high stromal NNMT had lower DSS in CRC, colon cancer and rectal cancer, as shown in Figure S2, and only had lower DFS in rectal cancer. As for stage III CRC patients, high stromal NNMT expression was also associated with poorer DSS compared with low stromal NNMT expression (Figure S3).

### 3.5 Stromal NNMT expression predicts an unfavorable survival in chemotherapy CRC

**Figure 4:**
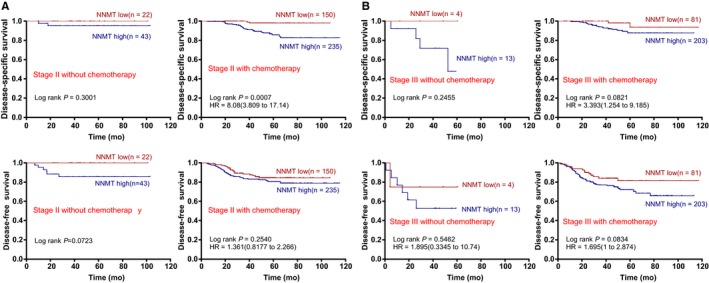
High stromal NNMT expression predicts an unfavorable survival in chemotherapy CRC. A, Associations between stromal NNMT expression and survival of stage II CRC patients with or without chemotherapy. B, Associations between stromal NNMT expression and outcomes in the stage III CRC patients with or without chemotherapy. Kaplan‐Meier survival curves of NNMT‐high (green line) and NNMT‐low (blue line) patients with stage II and stage III CRC who did or did not receive postoperative adjuvant chemotherapy are shown. *P*‐values and HRs are from Kaplan‐Meier analysis with log‐rank test

Next, the relationship between the stromal NNMT expression level and survival outcomes among patients with or without chemotherapy was investigated. For the stage II CRC patients who received chemotherapy, patients with high stromal NNMT had a shorter DSS but not DFS than those patients with low stromal NNMT, as shown in Figure 4A. For the stage III CRC patients who received chemotherapy, patients with high stromal NNMT had a shorter DSS and DFS than those patients with low stromal NNMT but with marginally significance (both *P* = .080), as shown in Figure 4B. However, the differences were not found in the stage II and the stage III CRC patients without chemotherapy (Figure 4), which may be associated with the small sample size in stage III CRC patients.

## 4 DISCUSSION

Recently, NNMT was reported as a master metabolic regulator of CAFs in ovarian cancer.^4^ However, the significance of NNMT in CRC stromal compartments is unknown. In this study, we applied a genomic data‐mining strategy to explore the compartments of NNMT expression in CRC. In our coexpression analysis of the genes, the results showed that the expression of NNMT mRNA was highly positively correlated with several known markers of CAFs, such as ACTA2 (also known as α‐SMA), VIM, TAGLN, FAP, and POSTN. In patient‐derived xenografts (PDXs), human stromal cells are replaced by mouse cells, allowing the differentiation of cancer and stromal cell gene expression based on the source of the transcript.^22^ Thus, we compared the expression of NNMT in primary tumors with that in PDXs and found that there was a fivefold reduction in PDXs. The evidences above strongly indicated that NNMT may be mainly expressed in stromal compartments of CRC and may be a potential CAF marker in CRC. Interestingly, the hypothesis was further confirmed by using IHC examination of CRC FFPE specimens. Therefore, we next focused on the clinical significance of stromal NNMT of CRC.

When comparing the expression between cancerous and noncancerous tissues from colorectal tissues, we found that stromal NNMT expression was significantly elevated in CRC tissues but there were no differences between primary CRC and liver metastasis CRC. Importantly, the expression of stromal NNMT was gradually increased among normal, adenomas and cancerous tissues. It has been reported that CAFs in CRC have the important roles in all the stages of CRC development and progression.^16^ In addition, the high expression of stromal NNMT was also associated with advanced TNM stage in our study. The results above strongly suggest that stromal NNMT plays an important role in the progression of CRC. With the exception of a recent study in ovarian cancer,^21^ previous studies have not clarified the relationship between stromal NNMT expression and progression of cancer patients. With a cut‐off value of 106 for the IHC‐score of stromal NNMT, we classified 967 CRC patients into two subgroups with high or low stromal NNMT tumors. The results demonstrated that patients with high stromal NNMT had a shorter DFS and/or DSS in CRC, which was consistently existed even if the cohort was divided into colon cohort and rectum cohort. The progression of cancer usually depends on tumor stage and grade, we firstly balanced the factors such as TNM stage and found that high stromal NNMT expression still independently predict an unfavorable outcome in CRC. These results strongly proved that stromal NNMT is a novel independent predictor of CRC outcomes.

Although the prognosis of early‐stage CRC patients is generally good, many patients may still relapse and develop distance metastasis.^23^ Ongoing efforts have been made to identify prognostic biomarkers for classifying patients with early‐stage CRC. For the stromal NNMT, we found that tumors with high stromal NNMT could predict the unfavorable outcomes in both early colon cancer and early rectal cancer. The results demonstrated that the stromal NNMT may be a potential prognostic marker in early CRC, but this finding still needs to be verified in other cohorts. Chemotherapy is a common treatment for patients with stage III CRC or a part of patients with stage II CRC^24^; however, the outcomes are unpredictable because of the heterogeneity of the CRC response to chemical drugs.^25^ The identification of the predictive biomarkers associated with chemotherapy is still ongoing. In this study, we found that the high stromal NNMT tumors are associated with a poor survival outcome only in the patients with chemotherapy but not in the patients without chemotherapy, which indicated that stromal NNMT may contribute to the resistance of chemotherapy.

With a large study population, we effectively evaluated the prognostic value of stromal NNMT in CRC. Nevertheless, the current study also has a few limitations. First, we cannot rule out the possibility of selection bias because of loss of follow‐up. Second, some important prognostic factors, such as microsatellite instability and extramural venous invasion,^26^ were not included, leading to incomplete inclusion of variables in multivariate Cox analysis. In summary, our study first evaluated the clinical significance of stromal NNMT in CRC and suggests that stromal NNMT is a prognostic marker in CRC and a potential predictive biomarker for chemotherapy. Furthermore, the biological function of stromal NNMT in CAFs remains to be further studied, and the prospective studies are needed to better understand stromal NNMT expression as a novel prognostic marker in CRC.
