# Supplementary material for: High stromal nicotinamide N‐methyltransferase (NNMT) indicates poor prognosis in colorectal cancer
Source: Cancer Med. 2020 Jan 27;9(6):2030–8. doi: 10.1002/cam4.2890 (PMC7064029; doi:10.1002/cam4.2890)
Supplement: Supplementary file 1 [file CAM4-9-2030-s001.docx]

**Supplementary Figure1**

**Supplementary Figure2**

**Supplementary Figure3**
