# Supplementary material for: High stromal nicotinamide N‐methyltransferase (NNMT) indicates poor prognosis in colorectal cancer
Source: Cancer Med. 2020 Jan 27;9(6):2030–8. doi: 10.1002/cam4.2890 (PMC7064029; doi:10.1002/cam4.2890)
Supplement: Supplementary file 2 [file CAM4-9-2030-s002.docx]

TableS1. Cox regression analysis of immunohistochemistry NNMT expression and clinicopathological covariates in the colon cancer patients

|  | **Disease-specific survival** | | | | |  | **Disease-free survival** | | | |
| --- | --- | --- | --- | --- | --- | --- | --- | --- | --- | --- |
|  | Univariate analysis |  |  | Multivariate analysis |  |  | Univariate analysis |  | Multivariate analysis |  |
| **Variables** | HR (95% CI) | *P* value |  | HR (95% CI) | *P* value |  | HR (95% CI) | *P* value | HR (95% CI) | *P* value |
| Age |  |  |  |  |  |  |  |  |  |  |
| >60 *vs.* ≤60 | 0.842 (0.460-1.541) | 0.577 |  |  |  |  | 1.114 (0.745-1.665) | 0.599 |  |  |
| Sex |  |  |  |  |  |  |  |  |  |  |
| Male *vs.* Female | 0.677 (0.363-1.262) | 0.219 |  |  |  |  | 0.848 (0.567-1.269) | 0.424 |  |  |
| Differential grade |  |  |  |  |  |  |  |  |  |  |
| (Well+Moderate) *vs.* Poor | 0.550 (0.133-2.277) | 0.410 |  |  |  |  | 0.986 (0.478-2.033) | 0.970 |  |  |
| Lymph nodes, n (%) |  |  |  |  |  |  |  |  |  |  |
| <12 *vs.* ≥12 | 2.394 (1.044-5.488) | **0.039** |  | 2.531 (1.108-5.783) | **0.028** |  | 2.719 (1.473-5.021) | **0.001** | 2.602 (1.396-4.848) | **0.003** |
| TNM stage |  |  |  |  |  |  |  |  |  |  |
| I+II *vs.* III | 1.643 (0.896-3.013) | 0.108 |  |  |  |  | 2.109 (1.413-3.147) | **<0.001** | 1.791 (1.164-2.758) | **0.008** |
| Chemotherapy |  |  |  |  |  |  |  |  |  |  |
| Yes *vs.* No | 1.717 (0.606-4.866) | 0.309 |  |  |  |  | 2.111 (1.018-4.377) | **0.045** |  |  |
| Serum CEA (ng/mL) |  |  |  |  |  |  |  |  |  |  |
| <5 *vs.* ≥5 | 1.740 (0.949-3.189) | 0.073 |  |  |  |  | 1.464 (0.983-2.180) | 0.061 |  |  |
| Serum CA199 (U/mL) |  |  |  |  |  |  |  |  |  |  |
| <37 *vs.* ≥37 | 1.477 (0.706-3.087) | 0.300 |  |  |  |  | 1.882 (1.193-2.968) | **0.007** |  |  |
| NNMT |  |  |  |  |  |  |  |  |  |  |
| Low *vs.* High | 6.337 (1.957-20.517) | **0.002** |  | 6.577 (2.031-21.303) | **0.002** |  | 1.484 (0.941-2.340) | 0.090 |  |  |

TableS2. Cox regression analysis of immunohistochemistry NNMT expression and clinicopathological covariates in the rectal cancer patients

|  | **Disease-specific survival** | | |  |  | **Disease-free survival** | | | |
| --- | --- | --- | --- | --- | --- | --- | --- | --- | --- |
|  | Univariate analysis |  | Multivariate analysis |  |  | Univariate analysis |  | Multivariate analysis |  |
| **Variables** | HR (95% CI) | *P* value | HR (95% CI) | *P* value |  | HR (95% CI) | *P* value | HR (95% CI) | *P* value |
| Age |  |  |  |  |  |  |  |  |  |
| >60 *vs.* ≤60 | 1.083 (0.558-2.102) | 0.814 |  |  |  | 0.821 (0.542-1.244) | 0.352 |  |  |
| Sex |  |  |  |  |  |  |  |  |  |
| Male *vs.* Female | 1.129 (.0573-2.221) | 0.726 |  |  |  | 0.922 (0.600-1.418) | 0.712 |  |  |
| Differential grade |  |  |  |  |  |  |  |  |  |
| (Well+Moderate) *vs.* Poor | 2.349 (0.910-6.064) | 0.078 |  |  |  | 2.792 (1.579-4.937) | **<0.001** | 2.250 (1.256-4.030) | **0.006** |
| Lymph nodes, n (%) |  |  |  |  |  |  |  |  |  |
| <12 *vs.* ≥12 | 2.633 (0.972-7.134) | 0.057 |  |  |  | 3.891 (1.761-8.596) | **0.001** | 3.601 (1.624-7.986) | **0.002** |
| TNM stage |  |  |  |  |  |  |  |  |  |
| I+II *vs.* III | 1.576 (0.807-3.080) | 0.183 |  |  |  | 2.453 (1.632-3.715) | **<0.001** | 2.108 (1.387-3.204) | **<0.001** |
| Chemotherapy |  |  |  |  |  |  |  |  |  |
| Yes *vs.* No | 0.769 (0.291-2.030) | 0.596 |  |  |  | 1.331 (0.662-2.676) | 0.422 |  |  |
| Serum CEA (ng/mL) |  |  |  |  |  |  |  |  |  |
| <5 *vs.* ≥5 | 1.398 (0.710-2.751) | 0.332 |  |  |  | 1.705 (1.127-2.577) | **0.011** | 1.606 (1.057-2.439) | **0.026** |
| Serum CA199 (U/mL) |  |  |  |  |  |  |  |  |  |
| <37 *vs.* ≥37 | 1.392 (0.540-3.592) | 0.493 |  |  |  | 2.032 (1.212-3.404) | **0.007** |  |  |
| NNMT |  |  |  |  |  |  |  |  |  |
| Low *vs.* High | 5.353 (1.639-17.486) | **0.005** |  |  |  | 1.885 (1.147-3.096) | **0.012** |  |  |
